# Comparative and Quantitative Global Proteomics Approaches: An Overview

**DOI:** 10.3390/proteomes1030180

**Published:** 2013-10-11

**Authors:** Barbara Deracinois, Christophe Flahaut, Sophie Duban-Deweer, Yannis Karamanos

**Affiliations:** 1 Université Lille Nord de France, Lille F-59000, France; E-Mails: barbara.deracinois@univ-artois.fr (B.D.); christophe.flahaut@univ-artois.fr (C.F.); sophie.duban@univ-artois.fr (S.D.-D.); 2 Université d’Artois, LBHE, Lens F-62307, France; 3 IMPRT-IFR114, Lille F-59000, France

**Keywords:** proteomics, proteomics: methods, electrophoresis, proteins and peptides, isotope labelling, fluorescent dies

## Abstract

Proteomics became a key tool for the study of biological systems. The comparison between two different physiological states allows unravelling the cellular and molecular mechanisms involved in a biological process. Proteomics can confirm the presence of proteins suggested by their mRNA content and provides a direct measure of the quantity present in a cell. Global and targeted proteomics strategies can be applied. Targeted proteomics strategies limit the number of features that will be monitored and then optimise the methods to obtain the highest sensitivity and throughput for a huge amount of samples. The advantage of global proteomics strategies is that no hypothesis is required, other than a measurable difference in one or more protein species between the samples. Global proteomics methods attempt to separate quantify and identify all the proteins from a given sample. This review highlights only the different techniques of separation and quantification of proteins and peptides, in view of a comparative and quantitative global proteomics analysis. The in-gel and off-gel quantification of proteins will be discussed as well as the corresponding mass spectrometry technology. The overview is focused on the widespread techniques while keeping in mind that each approach is modular and often recovers the other.

## 1. Introduction

The ability of detecting significant differences between two cellular states is a universal approach to unravelling the cellular and molecular mechanisms involved in a process with an ultimate goal of discovering new markers, diagnostics and indirectly to track new therapeutic routes. Cellular states are of physiological or pathological nature that may or may not be stimulated by an exogenous molecule exist in a changing environment, *etc*.

By carrying out the major portion of the cell functions, proteins play a major role in living organisms and are closely related to the phenotype of the cells. The word *proteome*, first used by Wilkins in 1994 [1], refers to the entire set of proteins including the modifications made on them, produced by a tissue or an organism, varying with time and under given physiological (or pathological) conditions. The analysis of a proteome, *proteomics* [2,3] can be applied to the study of proteins present in various types of biological materials, in particular to identify their functions and structures, for example the identification of interaction sites or PTMs. While the analyses are essentially performed with cells and/or tissues, the body fluid profiling was anticipated a few years ago [4] and seems to have a great future. The proteins display a large dynamic range between low and high abundance (1–10^5^ or 10^6^) and even larger in plasma (up to 10^9^–10^10^) [5].

**Figure 1:**
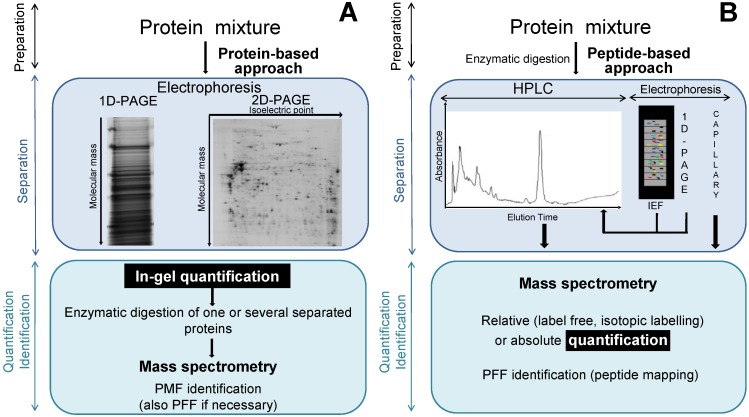
Flowchart of the most currently used techniques in view of a comparative and quantitative proteomics approach using a protein-based approach (**panel A**) or a peptide-based approach (**panel B**). The proteomic analysis is made up of four steps: (i) sample conditioning (not illustrated); (ii) sample preparation; (iii) separation; and (iv) quantification and identification of the proteins. The separation can be performed on proteins or peptides, by electrophoresis or chromatography. The quantification is possible either *in-gel* or *off-gel*, whereas the identification is always performed by MS. MS, mass spectrometry; HPLC: high performance liquid chromatography; IEF: isoelectric focusing; PAGE: polyacrylamide gel electrophoresis; PMF: peptide mass fingerprint; PFF: peptide *fragmentation fingerprint*.

The correlation between mRNA and protein levels is far from perfect [6] and certainly insufficient to predict protein expression levels from quantitative mRNA data [7]. No method, equivalent to PCR used for nucleic acids, is currently available for the amplification of proteins. Add to that, in proteomics, no method is able, in one step, to identify and quantify a complete set of proteins in a complex sample. A proteomics approach is a four key-step analytical process. The first step is dedicated to the cell or sample conditioning (cell growth conditions, cell collection, cell storage, cell disruption). The second step corresponds to the sample preparation (extraction, concentration, purification to remove contaminants such as lipids or nucleic acids, and storage of proteins) while the third is related to methods of separation, and the fourth to quantification and identification of proteins [8] (Figure 1).

Sample preparation is the most important step in order to obtain the right, reliable and reproducible result. Ideally the preparation should allow solubilisation of all the proteins in a sample, without any chemical modification, while eliminating all the interfering compounds (nucleic acids, polysaccharides, polyphenols, lipids, *etc.*) and remaining compatible with further analytical methods. Unfortunately, no universal protocol exists for the sample preparation although several protocols were adapted according to the biological sample and the objectives of the study [9].

The separation step can be carried out directly on proteins or on the set of peptides derived from the enzymatic digestion of the corresponding proteins. The separation of proteins or peptides can be considered in two ways: a first approach, “in-gel”, based on electrophoresis and, a second, “off-gel”, based essentially on chromatography. The most used methods for a global differential proteomics study remain the two-dimensional electrophoresis (2-DE) for intact protein-based profiling (Figure 1A) and HPLC for peptide-based profiling [10] (Figure 1B).

The quantification of proteins is conceivable for both aforementioned approaches. The use of radioisotopes as tracers is a technique that has been historically used for protein quantification. However, despite its high sensitivity, the use of radioisotopes have several drawbacks, in particular the high cost and the restrictive rules for their management due to the specific risk of radioactivity. Thus, recently other types of tracers emerged for the quantification methods. The in-gel quantification can be performed by measuring the colour intensity after fixation of dyes to the proteins while the off-gel quantification is always performed by MS. To that end proteins or the corresponding peptides can be directly analysed in MS (label free) or labelled by stable isotopes before MS-analysis.

Whatever the proteomic approach used, the identification of proteins/peptides is always carried out by MS. In addition to in-gel and off-gel approaches, two strategies were evidenced over the years. They are based on the way of identifying the proteins of interest and on the degree of information required for those proteins. The *bottom-up* strategy is historically the oldest and lies on the MS-analysis of peptides resulting from the enzymatic digestion of proteins. This strategy allows mainly the identification of proteins. More recent, the *top-down* strategy is based on the MS analysis of entire proteins [11]. The latter is a targeted approach allowing the identification of proteins but especially more comfortably characterisation of isoforms, post translational modifications (PTMs) or conducting of structural studies. Nevertheless, it needs significant amounts of biological samples as well as the separation and isolation of intact proteins. Consequently, the strategy of choice for a global differential study of proteins is clearly the *bottom-up* strategy.

This review will highlight the different techniques of separation and quantification of proteins and peptides in view of a comparative and quantitative global proteomics analysis. Only the most currently used techniques, precluding the radioisotopes, will be addressed. The reader can refer to a recent book which gives a detailed survey of the quantitative methods in proteomics [12].

## 2. In-Gel Quantification of Proteins

### 2.1. Gel Electrophoresis Techniques for Proteomics

Electrophoresis, conceived at the end of the 19th century [13], has continuously evolved over time, especially for biomolecules [14,15], and is now widely used to separate biological macromolecules and especially proteins that differ in size, charge and conformation. Three principles of electrophoresis have been described: (i) the zone electrophoresis, where the pH of the buffer conducting the current (and therefore the electrical field) remains constant throughout the electrophoresis time; (ii) the IEF that needs a pH-gradient to separate molecules and (iii) the isotachoelectrophoresis which consists, thanks to a current gradient, of an ordering of molecules according to their electrophoretic mobility rather than a real molecular separation.

Gel electrophoresis for proteomics uses a porous polyacrylamide supporting medium in which the proteins migrate according to their physicochemical properties in an electrolytic medium conducting the current and under the influence of an electric field. The protein electrophoretic mobility depends not only on the charge-to-mass ratio, but also on the physical shape and size of proteins. The proteins in a sample can thus be, more or less, separated from each other. Thanks to its adequate resolution and its low cost, the sodium dodecyl sulphate polyacrylamide gel electrophoresis (SDS-PAGE) is the technique of choice when only the identification of proteins is required. This most widely used electrophoresis method separates the proteins according to their molecular mass (MM) [16,17]. Indeed, due to its physicochemical properties, SDS binds non-covalently to proteins and brings them a constant electrical charge (1.4 g of SDS per g of protein) at pH > pKa of the SDS sulfonic group [18]. Therefore, all proteins display an identical charge density, and their electrophoretic mobility only depends on their MM. This technique is suitable for pre-purified samples or for samples with reduced complexity but in this case it can only provide a control of the sample composition. It can also serve as a pre-fractionation step for very complex samples.

The two-dimensional polyacrylamide gel electrophoresis (2D-PAGE) separates proteins in two steps, namely, an in-gel IEF of proteins to separate them according to their isoelectric point (*pI*), and a SDS-PAGE to separate proteins according to their MM [19,20]. This technique giving two dimensions of separation has a better resolving power and is therefore suitable to the analysis of complex samples. More than 2,000 spots can be resolved with gels of the highest resolution. The proteins are almost isolated from each other as spots thus allowing an easier and accurate identification. 

The tris-glycine discontinuous buffer system, termed “Laemmli’s system” [21], is the most widely used. This system uses two different buffers, differing in ion composition and pH—one for the gel and the electrode reservoirs—serving for the concentration of proteins in a stacking gel, and a second in a separating gel (thanks to the presence of leading and trailing ions). Several versions of this electrophoresis system have been developed and are adjustable to improve protein separation of particular samples when the “classic” electrophoresis has been proven to be insufficient [9]. Polyacrylamide gradients (low (up)-to-high (down) reticulation) can be used in PAGE in order to enhance the gel resolving power over a wider protein MM range. Concomitantly or separately, it is also possible to modulate the nature of the buffer ions and the pH of the buffer. Several buffer systems coexist depending of their leading and trailing ions. In the case of the tris-glycine buffer system, chloride plays the role of leading ion, whereas glycinate of trailing ion. However, other ions like acetate or MES, MOPS and Tricine can be used as leading or trailing ions, respectively (Bis-Tris; Tris-acetate or Tris-Tricine buffer systems). These different ion compositions offer different gel patterns and stability. The separation is suitable for larger or smaller proteins. The pH-lowering of the separation gel buffer will influence the charge of the buffer ions conducting the current, and therefore, the speed of the mobile fraction. The resolution of proteins of high MM will increase, but at the cost of a decreasing of the resolution of low MM proteins and vice-versa. It was shown that the yield of proteins recovered after 2D-PAGE, ranges between 25% and 50% [22]. In fact, some proteins tend to be insoluble, especially hydrophobic proteins, in the IEF experimental conditions and thus are entrapped in the IEF gel. Proteins are also lost into the buffers during equilibration prior to running in the second dimension run. Non-covalent and covalent labelling are currently used for the detection of proteins [23]. Those stains differ by their sensitivity, their linearity, their homogeneity and their MS-compatibility.

### 2.2. Post-Electrophoresis Staining of Proteins for their In-Gel Quantification

The protein spots can be detected after electrophoresis by direct *in-gel* staining (for review see [24,25]). Two of the most commonly used general protein stains are Coomassie brilliant blue and silver nitrate. Other techniques based on fluorescence are also available. In acidic solution, Coomassie brilliant blue (textile dye G250 and R250 mainly) binds to the basic and aromatic amino acids of proteins through electrostatic and hydrophobic interactions [26]. The Coomassie brilliant blue staining has a moderate sensitivity, at the ng level, with a good linearity and accuracy. The dye is not covalently bound and a conventional de-staining based on the use of organic solvents allows recovering intact proteins and compatible with their MS-analyses. The silver staining, at the pg level, is much more sensitive than Coomassie brilliant blue [27,28] but displays less good linearity and accuracy and is poorly adapted for MS analyses, since proteins can be covalently cross-linked when formaldehyde is used as reductant. This staining involves binding to the proteins of silver salts which precipitate after reduction as metallic silver [29]. A compromise should be found between the time of reaction of silver nitrate with proteins (on the gel surface) and the colouring intensity that will allow the analysis by MS from the protein amount remained intact in the central part of the gel. In addition, the amount of formaldehyde for the reduction of silver salts should be decreased to a minimum in the staining solutions and glutaraldehyde should be definitively avoided because of the irreversible protein nitrogen (and also other atoms) reticulation caused by these reagents. Silver nitrate staining is also sensitive to a number of external factors such as the temperature and the development time making the Coomassie brilliant blue staining the preferred staining for proteomics. It is also possible to stain the proteins by using organic fluorescent dyes (such as Deep Purple^TM^, a fluorescent dye based upon the natural compound epicocconone, originally isolated from the fungus *Epicoccum nigrum* [30], Flamingo^TM^ (Bio-Rad) and Krypton^TM^ (Pierce) and metal complex or metal chelates dyes (such as SYPRO Red and Orange [31], the well-known being SYPRO Ruby [32], RuBPS [33], ASCQ_Ru [34]) and IrBPS [35]). This fluorescent staining is sensitive (ng to pg level), non-covalent (or reversible for epicocconone) and, consequently, compatible to MS. Furthermore the quantification of PTMs (phosphorylation and glycosylation) is possible thanks to fluorescent labelling of the proteins at their phosphorylation (ProQdiamond) or glycosylation (ProQemerald) sites (*Multiplexed Proteomics*) [36,37]. Very recently it was shown that more sensitive, quantitative in-gel protein staining can be achieved [38] using an optimised protocol of the Neuhoff’s formulation of colloidal Coomassie brilliant blue [39]. In another method for the UV detection of proteins, trihalo compounds are included in the gel composition and react with tryptophan residues to produce fluorescence [40]. Whatever the staining method used, digitalised images of the gels, obtained by laser-based detectors, CCD camera systems and flatbed scanners, should be analysed with dedicated software [24]. The choice of imaging system largely depends on the type of protein dyes used. One of the constraints of the in-gel approaches is the variability found between gels. The low reproducibility is due to the more or less different electrophoretic migrations known as gel-dependent. Therefore, a differential in-gel approach needs an increased number of images to ensure an accurate and statistically reliable comparison.

### 2.3. Pre-Electrophoresis Staining of Proteins for their In-Gel Quantification

The *Difference gel electrophoresis* (DIGE) is a modification of 2D-PAGE that needs only a single gel to detect differences between two protein samples. This is done by fluorescent tagging of protein samples by different cyanine-based dyes before the electrophoresis step. The amine reactive dyes used should not modify the relative mobility of proteins common to the samples under investigation [41]. In the «minimal» labelling method, the fluorescent labelling reagent (*N*-hydroxysuccinimidyl ester cyanine dyes 2, 3 or 5; Cy2, Cy3 or Cy5) will react with free amino groups (amino-terminus and ε-amino groups of lysine residues). Labelling reaction is optimized so that only 2%–5% of the total lysine residues are labelled. *In fine*, using a relatively high protein/fluorophore ratio, a single lysine residue per protein molecule will be labelled (and most of the proteins remain unlabelled). In the «saturation» labelling method, the fluorescent labelling reagent (thiol-reactive maleimide derivatives of Cy3 and Cy5) reacts with free thiol groups of cysteine residues (obviously the thiol-free proteins will not be labelled). All the cysteine residues are thus labelled and saturation labelling is therefore much more sensitive than the minimal one, as more dyes are covalently bound to proteins. The «saturation» labelling is particularly adapted to low abundance proteins (see [42] for details).

**Table 1:** Different methods used for the staining or labelling of proteins in view of in-gel quantification(Protein-based quantification) ^a^.

			Advantages	Drawbacks	Robustness for large scale analysis
**Pre-electrophoresis staining** (Proteins labelled before electrophoresis)	**Chromophore-based staining**	none			
	**Fluorophore-based staining**	DIGE (cyanine)	Great linearity, sensitivity and reproducibility; MS-compatible	Expensive	Yes
	**PTM-specific staining**	none			
**Post-electrophoresis staining** (Proteins revealed after electrophoresis)	**Chromophore-based staining**	Silver staining, Zinc, Copper (metal-based)	Great sensitivity	Low reproducibility, linearity, and accuracy; Low MS compatibility, influenced by external factors	No
		CBB, ‘blue-silver’ (organic dyes)	Reproducibility, good linearity, good accuracy, MS-compatible	Moderate sensitivity	Yes
	**Fluorophore-based staining**	Sypro^®^, RuBPs, ASCQ_Ru, IrBPS (metal chelates)	Very good reproducibility, good linearity, great sensitivity, non-covalent labelling	Expensive	Yes
		Deep Purple^TM^, Flamingo^TM^, Krypton^TM^ (Organic dyes)			
	**PTM-specific staining**	ProQdiamond, ProQemerald	Very good linearity, good sensitivity	Expensive	Yes

^a^ DIGE, Difference gel electrophoresis; PTM, post translational modifications; CBB, Coomassie brilliant blue; RuBPs, Ruthenium (II) tris (4,7-diphenyl-1,10-phenatrolin disulfonate); ASCQ_Ru, ruthenium complex ((bis(2,2'-bipyridine)-4'-methyl-4-carboxybipyridine-ruthenium-*N*-succidimyl ester-bis(hexafluorophosphate); IrBPS, biscyclometalated iridium(III) complexes with an ancillary bathophenanthroline disulfonate ligand.

Samples from two (or three) different cellular states (physiological or pathological) are labelled with one of the fluorescent labelling reagents then combined prior to their electrophoretic separation in a single gel. Thus, the problem of variability between gels is suppressed and the number of processed gels decreased. In addition, the precision of the method can be improved by the use of a third fluorescent labelling reagent, often Cy2, used for the labelling of an internal standard composed of equimolar amounts of two samples to be compared. After the electrophoretic separation, the fluorescence intensities, originating from the three different samples, are quantified by digitalisation using fluorescence scanner. The obtained digital images are stored as tagged image file format (TIFF) or equivalent and compared using dedicated software. The DIGE technique displays a very good detection sensitivity (ng to pg level), a high linear dynamic range and is perfectly compatible with MS but is the most expensive [43]. The different methods used for the staining or labelling of proteins or peptides in view of in-gel quantification were summarised in Table 1.

### 2.4. Advantages and Limits of the In-Gel Quantification of Proteins

The 2D-PAGE and 2D-DIGE analyses offer several advantages. They allow obtaining a final analytical image which is quantitative, reproducible and “frozen” and representative of the protein heterogeneity in the sample of interest. In addition, the protein diversity resulted from PTMs is conserved and can be studied by various techniques including MS.

**Figure 2:**
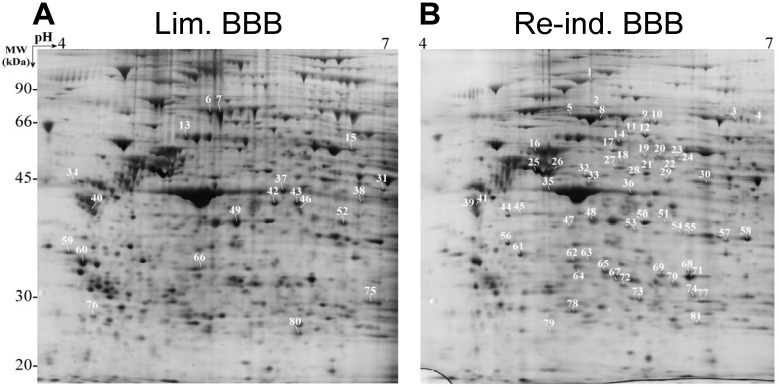
Comparison of the proteins extracted with Triton X-100 from bovine brain capillary endothelial cells showing limited (Lim. BBB) (**A**) or re-induced (Re-ind. BBB) BBB functionalities (**B**). Digital image obtained after 2D-PAGE of the proteins separated according to their *pI* and MW. The gel was silver nitrate stained. The numbering corresponds to the enriched protein in each condition. Each spot was identified by peptide mass fingerprinting (PMF) and/or peptide fragmentation fingerprinting (PFF) on a Proteineer ^TM^ workstation (adapted with permission from [45]).

The 2-DE has also some limits due to the particular physicochemical properties of a number of proteins, such as proteins of extreme MM (>200 kDa and <10 kDa), highly hydrophobic proteins, low abundance proteins (dynamic range of detection 10^3^ to 10^4^) or proteins of extreme *pI* (<3 or >11) [5,44]. In addition, despite the numerous technical optimisations during the electrophoresis steps, all the proteins of a given sample will not be revealed, thus leading to loss of information. Only the proteins revealed by staining are cut out and further analysed. Because the gel resolution is not always sufficient, the quantification of proteins is sometimes ambiguous, since one spot can contain more than one protein. Also, spot trains of proteins of equal molecular weight but different isoelectric points are commonly observed on the gels and mostly correspond to PTMs of proteins, such as phosphorylation and glycosylation. The immanent problem of 2D-PAGE, namely low resolution, multiple proteins for one spot and/or multiple spots for one protein (PTMs) is illustrated in Figure 2 showing a result obtained during the comparison of the proteins extracted with Triton X-100 from bovine brain capillary endothelial cells with limited (Lim. BBB) or re-induced (Re-ind. BBB) BBB functionalities. A representative sample among the proteins identified during this study [45] is displayed in Table 2. Internet-based prediction tools can be used for comparing the theoretical models to the actual gel pattern [46]. Finally, the analysis by electrophoresis is limited to the study of the more abundant proteins, which constitutes sometimes an advantage [47], and unsuitable to a high throughput screening. Nevertheless, 2-DE is not abandoned and the still existing importance of the two dimensional electrophoresis in the biology field was recently illustrated [48].

## 3. Off-Gel Quantification of Proteins (Peptide-Based Quantification)

In addition to the aforementioned approaches for the quantification of intact proteins after gel electrophoresis, the peptide-based quantification of proteins by MS (*shotgun* approach) is continuously evolving. The proteins in a sample are directly submitted to enzymatic digestion and the mixture of the resulted peptides, whose molecular mass ranges from 500–4,000 Da, are separated and subsequently analysed by MS. However, this approach is considerably limited for biological samples of high degrees of complexity, because of the increase in the complexity/heterogeneity of the sample due to the multiplication of molecular species generated by the enzymatic digestion. Pre-fractionation of the proteins prior to the proteolysis step or analysis of sub-proteomes can be proven in this case advantageous [49,50]. In this way, the sensitivity and the ratio of identifications are improved.

**Table 2:** A representative sample among the proteins identified during 2D-PAGE. The proteins were extracted with Triton X-100 from bovine brain capillary endothelial cells with limited (Lim. BBB) or re-induced (Re-ind. BBB) BBB functionalities. The Table illustrates the presence of one spot for one protein, multiple proteins for one spot and/or multiple spots for one protein. The identifications were done by PMF and PFF after MALDI-TOF/TOF mass spectrometry. Proteins over-abundant in Lim. BBB are highlighted in grey. The data were reproduced with permission from [45].

	Spot number	Protein Name	Swiss-Prot Accession	Theoretical		Experimental		PMF Mascot Score ^c^	Sequence Coverage (%)	Matched/ Unmatched peptides	Identification by MS or MS/MS	Number of fragmented peptides
				MW (kDa) ^a^	pI ^b^	MW (kDa) ^a^	pI ^b^					
**One spot for one protein**	45	Serine-threonine kinase receptor-associated protein	STRAP_BOVIN	38.4	4.99	40.5	4.90	242	67	21/25	MS	
	47	Inorganic pyrophosphatase	IPYR_BOVIN	32.8	5.27	39.7	5.09	168	42	12/14	MS	
	58	Phosphatidylinositol transfer protein alpha isoform	PIPNA_BOVIN	31.8	6.12	34.8	5.54	73	28	8/15	MS	
	72	6-phosphogluconolactonase	6PGL_BOVIN	27.6	5.57	29.9	5.44	129	40	10/19	MS & MS/MS	1
	78	Apolipoprotein A-I precursor (Apo-AI)	APOA1_BOVIN	30.2	5.71	26.2	5.52	366	68	25/16	MS	
	79	Sorcin	SORCN_HUMAN	21.7	5.32	24.1	4.98	153	48	15/28	MS	
**One spot for several proteins**	36	Actin, cytoplasmic 1 (Beta-actin)	ACTB_BOVIN	41.7	5.29	44.2	5.52	94	44	13/50	MS & MS/MS	1
		Succinyl-CoA ligase [GDP-forming] beta-chain, mitochondrial [Precursor]	SUCB2_BOVIN	46.7	7.51	44.2	5.52	112	28	14/49	MS/MS	1
	37	Actin, cytoplasmic 1 (Beta-actin)	ACTB_BOVIN	41.7	5.29	44.2	5.66	80	44	14/91	MS	
		Leukocyte elastase inhibitor	ILEU_BOVIN	42.2	5.70	44.2	5.66	134	49	22/83	MS	
		Succinyl-CoA ligase [ADP-forming] beta-chain, mitochondrial [Precursor]	SUCB1_BOVIN	50.0	6.73	44.2	5.66	100	44	20/85	MS	
**Several spots for one protein**	67	Chloride intracellular channel protein 4	CLIC4_BOVIN	28.7	5.6	30.9	5.42	159	68	19/47	MS	
	70	Chloride intracellular channel protein 4	CLIC4_BOVIN	28.7	5.6	30.2	5.69	288	81	22/14	MS	
	13	Vimentin	VIME_BOVIN	53.7	5.06	57.0	4.91	233	56	23/16	MS & MS/MS	1
	16	Vimentin	VIME_BOVIN	53.7	5.06	52.3	4.82	373	78	45/48	MS & MS/MS	3
	39	Vimentin	VIME_BOVIN	53.7	5.06	42.5	4.55	296	69	37/39	MS & MS/MS	1
	40	Vimentin	VIME_BOVIN	53.7	5.06	41.7	4.54	228	57	29/37	MS & MS/MS	1
	41	Vimentin	VIME_BOVIN	53.7	5.06	43.1	4.53	118	33	10/11	MS	
	64	Vimentin (Fragment)	VIME_BOVIN	17.2	9.92	32.2	5.16	89	27	11/19	MS	
	65	Vimentin	VIME_BOVIN	53.7	5.06	31.6	5.38	265	42	34/43	MS & MS/MS	4
	66	Vimentin	VIME_BOVIN	53.7	5.06	35.3	5.16	205	42	21/16	MS	
	76	Vimentin	VIME_BOVIN	53.7	5.06	26.7	4.50	110	32	12/20	MS & MS/MS	1

^a^ Molecular weight; ^b^ Isoelectric point; ^c^ Score correspond to the measures of certainty (*p*-values <0.05).

### 3.1. Reducing the Complexity of the Sample by Pre-Fractionation of Proteins

In order to reduce the complexity of the samples, the proteins can be separated by electrophoresis, chromatography, proteome fractionation based on differential solubility, and by aqueous two-phase system (ATPS). Electrophoresis can be performed by 1D-PAGE (see Section 2.1), or using a free-flow protein purification (*off-gel*) technique based on IEF [51]. The proteins of interest, in gel strips (1D-PAGE) or in solution (*off-gel*) are then further analysed. A pre-fractionation by protein thermal denaturation can increase the resolving power of 2D-PAGE [52]. The complexity of a proteome can be decreased by differential solubilisation (for review [53]) with increasing concentrations of acetonitrile [49,50], and by methods based on ATPS extractions [54]. Liquid chromatography methods, such as size-exclusion [55], ion-exchange [56], affinity [57], and reverse-phase chromatography [58] can also be used to fractionate the proteins (for review [59]). They are essentially useful for low complexity samples and are detergent sensitive. Thus, they should be mainly used as a means of enrichment of certain proteins of interest that display specific biochemical properties. 

### 3.2. Separation of Peptides

The use of electrophoresis for the separation of peptides according to their charge is mainly carried out by liquid phase migration in *off-Gel* IEF [60] and capillary electrophoresis (CE). IEF of peptides released from a mixture of proteins is generally performed in view of pre-fractionating the sample (like LC for proteins) to reduce its heterogeneity. The use of CE, which was used occasionally a few years ago for the separation of peptides and even proteins (top-down analysis) before their analysis by MS, is expanding and the CE-MS coupling is now functional and efficient with several types of mass spectrometers [61,62]. However, this coupling is recent and in spite of the higher sensitivity and higher resolution power of CE, it is still less used than HPLC, which is nevertheless a powerful tool in analytical chemistry, the reverse-phase (RP)-HPLC being the most used in proteomics. This trend is expected to reverse in the coming years.

In RP-HPLC, the peptides are separated according to their partition coefficient between a hydrophobic stationary phase, often C18 bonded silica, and an aqueous mobile phase whose polarity decreases by the application of a gradient of raising concentration of an organic water-miscible solvent, such as acetonitrile. Other separation modes of various selectivities can be used, such as ion exchange chromatography, exclusion chromatography, and chromatography based on hydrophilic or hydrophobic properties [63].

**Figure 3:**
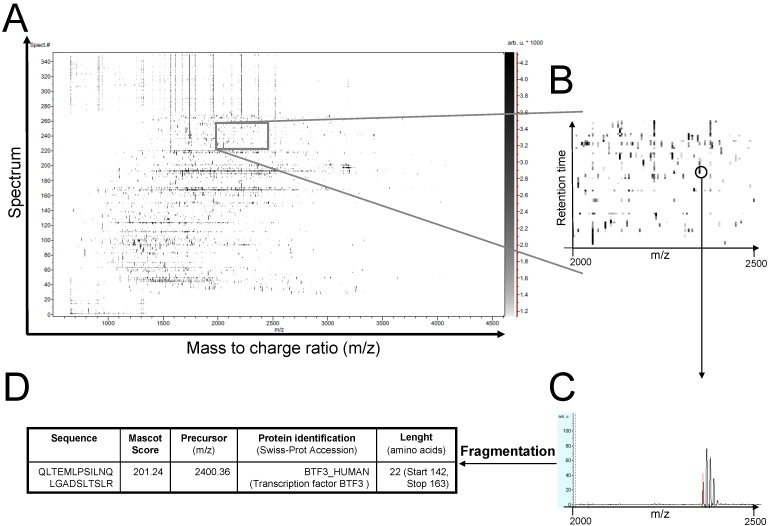
2D-like view illustrating the distribution of the peptides according to their retention time *vs.* their *m/z* ratio. (**A**) print-screen image obtained from Warp-LC Survey Viewer (Bruker Daltonics) after RP-HPLC-MALDI-TOF-MS analysis of tryptic peptides issued from the enzymatic digestion of a sample of proteins extracted from brain capillary endothelial cells; (**B**) detail of a particular region; (**C**) selection of an ion; (**D**) fragmentation and identification of the peptide (unpublished data from the authors’ laboratory).

Several factors influence the chromatographic separation of peptides, namely the characteristics of the column itself (length, granulometry, chemical nature and type—monolithic or not—of the stationary phase and internal diameter), the elution gradient (solvent’s concentration, flow and time) and the temperature. The peptides eluted during a chromatographic *run* can be either directly, *on-line*, analysed by MS using an electrospray source or indirectly, *off-line*, in the case of a MALDI source. HPLC is considered as a pre-fractionation step of peptides prior to MS and, “coupling” to MS, has proven to be an extremely efficient tool for the nearly-complete identification of proteins of a complex mixture. In a way, HPLC overcomes the limits of *in-gel* techniques but no visual illustrates the heterogeneity of the proteins of a given sample and no information on *pI* or MM is assessed by this way, thus making interpretation more difficult. In fact, 2D-PAGE electrophoresis affords the only visual illustration of the heterogeneity of proteins of a given sample (Figure 2). Nevertheless, data processing allows generating 2-D representations (retention time *vs.* *m/z* ratio) of the peptide separations to illustrate the distribution of all the injected and detected peptides (Figure 3). 

### 3.3. Peptide-Based Quantification of Proteins

For more complex samples, by analogy with 2D-PAGE, two (or more) chromatographic systems based on different selectivity can be coupled together before the MS analysis of separated peptides (2D-LC). This chromatography coupling offers two dimensions of separation and allows a high number of identifications, since the resolution power (corresponding to the number of compounds that have been separated) reflects the scaling of the resolution powers of the two chromatographic systems [64]. Usually, the last separation step, before MS is a RP-HPLC. However, during the first separation step, the vast majority of chromatographic methods can be used [65,66]. 

**Figure 4:**
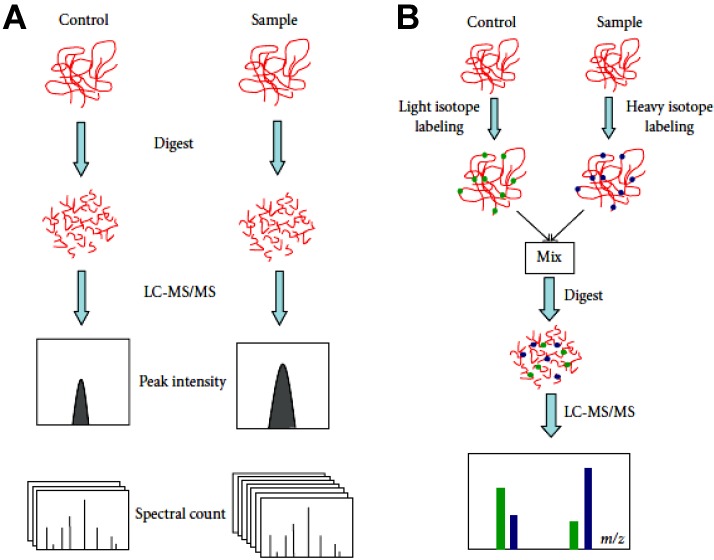
Differences between label free quantification (**A**) and quantification by means of stable-isotope labelling (**B**). The label free quantification consists of two analyses carried out independently before their comparison. The quantification by means of stable-isotope labelling allows the direct comparison of isotope-labelled peptide pairs. LC-MS/MS, *liquid chromatography coupled to tandem MS*. (Adapted from [69], Creative Common Attribution License CC-BY).

Quantification of proteins can be achieved by MS. Two approaches are currently used, namely, the label free quantification (Figure 4A) and quantification based on the preliminary labelling of the protein or the corresponding peptides (Figure 4B). The latter approach is undoubtedly the most diverse, due to the extremely great variety of covalent chemical labelling [67,68]. For the neophyte, this profusion of labelling methods makes the choice of the most convenient and adequate method difficult in the frame of a proteomics study that needs to be developed.

The label free quantification is the most widespread technique as it is inexpensive; however, it needs some corrections to be accurate [70]. MS in itself is not a quantitative tool. In fact, the peptides obtained by enzymatic digestion have a large range of physicochemical properties that induce a response in MS proper to each peptide. Under these conditions, in order to obtain accurate quantifications it is necessary to compare each peptide individually between the experiments. The MS quantification methods based on the use of stable isotopes, although expensive, are the most adapted to overcome the problem. Indeed a labelled peptide will be chemically identical to the native peptide leading to the same behaviour of these MS distinguishable peptides during the chromatographic step(s) and during MS analysis (visualisation of the isotopic profiles in a single spectrum). Different profiles can be observed in the case of deuterium isotopologues due to secondary isotope effects [71].

Almost all the quantification methods are relative, and concern the determination of the relative abundance via the ratio of intensity of ions of the corresponding “couple” of peptides. The absolute quantification is possible but today remains mainly a targeted approach; nevertheless the absolute quantification in the frame of global proteomics is developing [72].

#### 3.3.1. Relative Quantification

##### 3.3.1.1. Label Free Quantification

A quantitative differential proteomics approach has three important aspects, namely, rapidity, efficiency and reproducibility of analyses. The label free quantification is largely employed for its rapidity, its low cost and its simplicity of use. Two methods for the label free quantification are reported and widely reviewed [73,74]. In the first, based on the comparison of mass spectra, the change in the signal intensity of a peptide (area or peak intensity) is correlated to the protein quantity [75]. In the second, based on spectral counting, the number of peptides sequenced for a given protein is correlated to its quantity [76]. The label free quantification implies that the samples to be compared are prepared separately and individually analysed by MS/MS. The changes in terms of protein abundance are calculated after the comparison to other analyses.

The MS-signal intensity of a peptide detected in MS can be correlated to its quantity following its ionisation in electrospray ionisation mode [77]. The ion chromatograms for every peptide are extracted from a LC-MS/MS run and their peak areas integrated over the time scale. Extracted ion chromatograms (XIC) for mass to charge ratios are determined for each peptide. In this way, the MS-signal intensity of peptides in an experiment can be compared to the corresponding peptides in one or several other experiments thus giving information on their relative quantification [75]. The main drawback of this approach is the experimental variations of MS-signal intensities of peptides. An excellent reproducibility and a normalisation using internal standards and/or a normalisation coefficient are in this case mandatory to more accurately identify common peptides between different experiments. Dedicated software for the alignment of LC-runs take advantage of the retention times and the *m/z* of the signals to identify similar peptides [78]. The various types of software offer automation of the data analyses, which are manually unrealisable, considering the huge amount of data generated by such an approach. The intensity of peaks is computed and compared between the different chromatographic separations. Although minimised with high resolution mass spectrometers, sometimes very close mass signal interferences can occur, leading to quantification errors. Therefore, technical replicates are mandatory in order to proceed to a correct interpretation of data. A compromise should be found between the solidity of the quantification and the number of identified proteins. A first phase is dedicated to identifying a maximum number of proteins through fragmentation (MS/MS mode) and a second is focused on the improvement of MS-settings in order to optimise the MS-signals (resolved peaks) of peptides for their accurate quantification. 

After enzymatic digestion a high abundance protein will generate a large number of peptides. In the spectral counting *label-free* quantification method [76], the relative quantification of proteins is based on the comparison of the number of MS/MS spectra performed for a given protein between several experiments. The abundance of a protein is correlated to the number of MS/MS spectra leading to its identification. In this approach, the tandem MS mode gives the identification together with the quantification of a protein. This method is controversial since it does not take into account the physical properties of the peptides and assumes that the mass response is linear and identical for each protein, and yet the response in terms of spectral counting will be different for each peptide because the retention time and the chromatographic peaks’ width are different. Therefore, MS-signal normalisation as it pertains to the protein size and the statistical analysis of the obtained data is unavoidable in order to obtain accurate quantification.

The relative abundance of a protein can also be reported as the *protein abundance index* (PAI) [79] corresponding to the number of observed peptides (within the dynamic range of the mass spectrometer used) divided by the number of the observable peptides per protein. The exponential modified *protein abundance index* (emPAI), derived from PAI (it equals 10^PAI^-1), is proportional to protein content in a protein mixture [80]. The *absolute protein expression* (APEX) quantitative proteomics tool improves on basic spectral counting methods by including a correction factor (peptide detection probabilities depending on their physical properties and according to the mass spectrometer used) for each protein that accounts for variable peptide detection by MS techniques [81,82].

##### 3.3.1.2. Quantification Using Stable Isotope Labelling

The techniques of stable isotope labelling were introduced in 1999 [83,84,85]. The differentiation strategy is based on distinctive labelling of proteins or peptides, in each particular condition. Two peptides tagged with different stable isotopes (“couple of peptides” or isotopologues) that have the same physical properties will undergo the same ionisation and will be detected in the same spectrum with a mass shift depending on the isotope used. Quantification can be performed either by MS or tandem MS (MS/MS) depending on the objectives. It lies on the search and the quantitative comparison (area under curve or peak intensity), within the same chromatographic fraction, of signals corresponding to the isotopic pair of labelled and unlabelled peptides. A ratio is calculated to establish the relative quantity of each peptide. A ratio of 1 corresponds to an equal amount of each peptide while a ratio imbalance corresponds to the overabundance of one of the peptides. Included in the same mass spectrum, the signals need no retention time correction, unlike in the label free techniques.

The tag mass shifts, easily detected by MS, are integrated in the sophisticated algorithms of search engines and can be divided into three types: (i) labelling *in vitro/(or in vivo)* during cell culture (or growth) by the addition of the constitutive atoms or labelled amino acids in the growth medium (or foods); (ii) labelling of the proteins after their extraction from the cells; and (iii) labelling of the peptides over or after the enzymatic digestion of the proteins before their analysis. The tag can be introduced metabolically (for the 1st case), chemically (2nd and 3rd case) or enzymatically (3rd case). Several stable isotopes can be used: carbon-13 (^13^C), hydrogen-2 (^2^H or D, deuterium), oxygen-18 (^18^O) or nitrogen-15 (^15^N). The terms of light (^12^C, ^1^H, ^16^O, ^14^N) and heavy (^13^C, ^2^H, ^18^O, ^15^N) isotopes are used. 

###### 3.3.1.2.1. Metabolic Labelling

The metabolic labelling is carried out during protein synthesis. Although now developed for *in vivo* animal experimentation, this method has been historically utilized for *in vitro* quantitative proteomics. The cells should be cultivated in a normal, non-isotopic medium or in a medium exclusively enriched with the heavy isotope. After adequate cellular multiplication, the cell division, cell growth and all the cellular processes lead to the total incorporation of the isotope in the proteins of the cultivated cells. The cells of both conditions are combined and further treated as a single sample. The labelling is performed early in the protocol, thus reducing the experimental bias introduced when the incorporation of the tag is performed late in the experimental process. Total incorporation of the label is a decisive point for the quantification and should be accurately checked by MS. It is considered as total when no unlabelled peptide is observed for the “heavy medium” condition. Therein, several cell divisions are necessary depending on the nature of the cells used. The relative amount of proteins is determined by the ratio of MS-signal intensities of peptide pairs. The identification is performed by MS fragmentation of the peptides. This technique is ideally used for cell lines; especially the immortalised cell lines admitting several passages, however, it is lengthy and expensive. Two types of metabolic labelling are available, namely, labelling with ^15^N or ^13^C and labelling by amino acid isotopes in the culture medium. However, classical methods for PTMs remain in use.

The cells can be grown in isotopically enriched media, for example in ^15^N-labelled growth media, or in the unlabelled (^14^N) counterpart [84]. After several cycles of incorporation, the cells contain proteins with labelled amino acids thanks to the *de novo* synthesis. Alternative stable isotopes, such as ^13^C and ^18^O, could be used in the present context. The negative aspect of this technique is that the mass difference between the labelled and the corresponding unlabelled peptides will depend on the amino acid sequence (number of N, C or O) thus complicating the data analysis. Moreover, this strategy is clearly inadequate for organisms that do not directly incorporate N, C or O.

Introduced in 2002, the *stable isotope labelling by amino acids in cell culture* (SILAC) technique overcomes this last point by the substitution of one or two amino acids of the growth medium by their isotope-labelled counterparts [86]. Essential amino acids, or those present in high concentration in the medium, should be used in preference, to avoid their *de novo* synthesis by the cells and should be detected in as many peptides as possible Moreover, the selected amino acids should not be metabolised in the cells to other amino acids, because this could encounter additional labelling making the data analysis difficult. For example arginine is converted to proline in some cell lines. This pitfall can be avoided by adding excess proline [87]. Leucine and lysine, and also tyrosine and arginine are the most used amino acids for SILAC [88]. A double labelling, for example Arg/Lys (^12^C_6_/^13^C_6_-arginine/^12^C_6_/^13^C_6_-lysine) is also conceivable to ensure an almost ideal labelling of all the peptides (except the C-terminus tryptic peptide of the protein) when trypsin is used for the digestion. In that way, uncleaved peptides will be double labelled. Up to five different cellular states were compared using such a multiplex SILAC-based strategy [89]. 

In the *culture-derived isotope tags* (CDITs) technique which is derived from SILAC [90], a labelled internal standard is generated for quantifying the proteome of a given tissue. This standard corresponds to cells originated from the tissue under consideration, grown in a stable isotope-enriched medium according to the SILAC technique. For example, cells of mouse neuroblastoma are mixed with each mouse brain sample. For each brain sample, the ratio between the isotopic distribution of peptides of same sequence (from the tissue to analyse and from the cultured cells) is determined (one ratio per sample). The change in the protein abundance between the two brain samples is then determined by the ratio of the two ratios previously calculated. Interestingly, a protein found in a brain sample but not in the cultured cells can also be quantified. The ratio of the target peptide, which does not have a corresponding labelled peptide in the standard cells, can be obtained by using the peak ratio against an isotope-labelled peptide of different sequence found in the standard cells and having the same retention time in LC/MS.

The super-SILAC technique, recently introduced [91,92] consists of adding a mixture of lysates of several cell lines labelled by SILAC as internal standard to proteomic samples to be analysed. This improvement is used for the characterisation and the comparison of cell lines, especially tumour cells.

The SILAC technology was also implemented for *in vivo* animal experiments [93,94] and is known as *stable isotope labelling of mammal* (SILAM). The animals are metabolically labelled with stable isotopes; their diet is exclusively composed of stable isotope-enriched proteins. Compared to *in vitro* labelling methods, metabolic labelling ensures that each protein is enriched with the heavy isotope, thus “all” the cells of those animals consist of labelled proteins. The animals receiving this labelling were shown to be healthy and phenotypically identical to the unlabelled ones [93], but their production is lengthy, as it requires several generations and is expensive.

Using these strategies of metabolic labelling, it is possible to identify and quantify PTMs like methylation (method named heavy methyl SILAC) [95]. In this case, the cells are grown in media containing [13CD3]-methionine allowing, after cell divisions, the labelling of all the *in vivo* methylation sites.

###### 3.3.1.2.2. Chemical and Enzymatic Labelling

Chemical or enzymatic labelling is applied later in the proteomic experimental process than the metabolic labelling. This type of labelling is therefore not limited to cell cultures and is applicable to all types of biological samples. It can concern a reactive group of a specific amino acid in the proteins or peptides (often the thiol group of cysteine or the ε-amino group of lysine residues) or the α-amino- or α-carboxyl terminal groups of peptides (Table 3). However, variations of labelling ratios could be due to protein isoforms. For example, histones bear PTMs (methylation, acetylation, botinylation, ubiquitinylation, *etc.*) on lysine residues and on the N-terminus that can hinder the labelling [96]. In this case the MS-signal ratio cannot indicate a difference of protein concentration, only the presence of PTMs. Furthermore, chemical labelling can cause secondary reactions giving unexpected products and, thus, influences the quantification results.

**Table 3:** Chemical and enzymatic labelling of proteins or peptides ^a^.

Reactive groups	Methods	Targets	Amino Acids	nb of samples	References
**Thiol**	ICAT	proteins	cysteine	2	[83]
	ALICE	proteins	cysteine	2	[97]
	Photocleavable-ICAT	proteins	cysteine	2	[98]
	**N**-ethymaleimide/iodoacetamide	proteins	cysteine	2	[99]
	acrylamide or vinylpyridine	proteins	cysteine	2	[100,101]
**Amino**	ICPL	proteins	N-term/Lys	2,3,4	[102,103]
	Post-digest ICPL	peptides	N-term/Lys	2	[104]
	iTRAQ	peptides	N-term/Lys	2,4,8	[105]
		/proteins			[106]
	TMT	peptides	N-term/Lys	2,6	[107]
	Dimethyl	peptides	N-term/Lys	2,4	[108,109]
**Carboxyl**	EMOS	proteins	C-term	2	[110,111,112]
		peptides	C-term	2	[113]
	AMOS	peptides	C-term	2	[114]
	Methanol	peptides	C-term/	2	[115]
			Asp/Glu		

^a^ ICPL, isotope-coded protein label; TMT, tandem mass tags; ICAT, isotope-coded affinity tags; iTRAQ, isobaric tags for relative and absolute quantification; ALICE, acid-labile isotope-coded extractants; EMOS, enzyme mediated oxygen substitution; AMOS, acid mediated oxygen substitution.

####### 3.3.1.2.2.1. Labelling of Thiol Groups

Developed in 1999 [83], the *isotope-coded affinity tags* (ICAT) labelling is obtained by alkylation of cysteine thiol groups with either a light or a heavy tag using iodo-acetamide groups conjugated to biotine by a spacer arm. The difference in mass is introduced on the spacer arm, which can contain proton or deuterium (H or D) (ΔDa = 8 Da) or alternatively ^12^C or ^13^C. This labelling is performed on entire proteins but can also be done on peptides. After enzymatic digestion, the cysteine-containing peptides are then purified by affinity chromatography (biotine-avidin/streptavidin systems) and analysed by LC-MS/MS, whereby they are detected as doublets in the same mass spectra, and their relative intensity reflects the relative abundance of the original protein in both samples. The overall heterogeneity of the samples is reduced, since only the cysteine-containing peptides are analysed, but the counterpart is that lower sequence coverage is obtained for a given protein. In addition, the study of PTMs is limited. An excess of the tag or the occurrence of endogenous biotine decreases the labelling efficiency and thus limits the use of the affinity chromatography step [83]. The biotine, which causes the decrease of the MS collision energy and then decreases the MS/MS fragmentation efficiency, can be removed before the MS step by the use of photocleavable linker or acid-cleavable linkers. Historically, the first acid-cleavable technique, derived from ICAT and termed *acid-labile isotope-coded extractants* (ALICE), contains (i) a thiol-reactive group (used to capture all cysteine-containing peptides from peptide mixtures and then eliminates the affinity chromatography step), (ii) an acid-labile linker, and (iii) a non-biological polymer [97]. Later, a generation of ICAT reagents with photocleavable linker was also developed [98], where the UV-light is used to liberate the biotine used during the purification step. Finally, ICAT reagents were worked out carrying an acid-cleavable bond to discard the biotine, after the affinity chromatography step [116]. Today, this last technique remains the most used. The use of stable-isotope labelling at thiol groups by small organic molecules like *N*-ethymaleimide (^1^H_5_-NEM/^2^H_5_-NEM, Δ = 5 Da) or iodoacetamide (^12^C_6_-IAA/^13^C_6_-IAA, Δ = 6 Da) was described in a first study combining one- or two-dimensional electrophoresis and MALDI-TOF-MS [99].

This method eliminates fundamental problems of the other existing isotope-tagging methods requiring liquid chromatography and MS/MS, such as isotope effects, fragmentation, and solubility. It is also being considered to be more practical and accessible than those LC-dependent methods. Another method is based on the differential labelling of mixtures by use of a commercially available, unlabelled and labelled with deuterium, acrylamide or vinyl pyridine (^1^H_3_/^2^H_3_-acrylamide or ^1^H_4_/^2^H_4_-vinylpyridine) [100,101] to alkylate proteins (cysteine residues). With the latter method, small, hydrophilic molecules have the advantage of improving the solubility of the proteins and easily accessing the labelling sites. After mixing the samples from the two conditions, the proteins are separated by two-dimensional (2-D) gel electrophoresis and analysed by mild MS-ionisation to prevent the fragmentation of the tag (often occurring in the ICAT technique) [101]. However, although this method has some of the 2D-DIGE advantages (e.g., several samples on a single gel) it also has the classical drawbacks of the *in-gel* analysis. The quantification is performed by MS instead of measuring the staining intensity of spots. The 2D-PAGE is used for the identification of the proteins by PMF. This simplifies the overall analysis and again prevents the fragmentation of the ICAT tags.

####### 3.3.1.2.2.2. Labelling of Amino Groups

Proteins can be covalently labelled on their free amino-terminal groups and especially on the ε-amino groups of lysine residues that are more abundant than cysteine residues in proteins. The disadvantage of this type of labelling, but to a lesser extent than the labelling on cysteine residues, is that it does not cover the entire protein and, thus, can induce loss of information. Nevertheless, it is possible to simultaneously tag the peptides at their carboxyl-terminal and amino-terminal extremities in order to obtain a uniform labelling of all the peptides regardless of their amino acid composition and their PTMs. Introduced in 2005, the *Isotope-coded protein label* (ICPL) approach [102] involves the labelling of amino groups. The tags are *N-*nicotinoyloxy-succinimide (^12^C_6_/^13^C_6_), whose the mass difference (ΔDa = 6.02 Da) is easily observable by MS. Quantification is performed in the MS mode, by the comparison of the peak intensity while the identification of proteins is classically done in the MS/MS mode. Recently, triplex and quadruplex versions of ICPL were developed using a combination of carbon and hydrogen isotopes (^12^C_6_/^1^H_4_, ^12^C_6_/^2^H_4_, ^13^C_6_/^1^H_4_, ^13^C_6_/^2^H_4_) for the simultaneous comparison of three or four samples [103]. This multiplex approach allows to reduce the proteome complexity (even after labelling), and to detect isoforms, PTMs and also splicing variants (through 2D-PAGE). After labelling, thanks to the low MM and the hydrophilic properties of ICPL-tags, the original physicochemical properties of proteins, especially their ability to precipitate in aqueous solutions, are preserved. In addition, the nicotinic derivative enhances the in-source ionisation of peptides (increase of MS-signal intensities) and promotes the MS fragmentation mechanisms (the MS-fragmentation is improved). The labelling of lysine residues prevents the digestion by trypsin which will only be effective at the arginine residues. The generated peptides are longer, thus their fragmentation is more difficult. This can be easily overcome by a double enzymatic digestion, for example a combination of trypsin/Glu-C endoproteinases in order to obtain shorter peptides. Initially designed for protein labelling, the ICPL can be performed directly on peptide mixtures. Using this *post-digest* ICPL, the amount of identified but non quantified proteins can decrease from 30% down to 2% [104].

The *isobaric tags for relative and absolute quantification* (iTRAQ) labelling are also based on the covalent labelling of amino groups of peptides [105]. Designed for peptides, this labelling method is less commonly used for proteins [106]. The label is composed of a reporter chemical group, a balance chemical group and a chemical group reactive on primary amines. Today, the iTRAQ technology allows comparing 2, 4 or 8 samples at once. The *reporter* groups are distinguished by their particular mass (113, 114, 115, 116, 117, 118, 119, 121 Da), according to the combinations ^12^C/^13^C, ^16^O/^18^O and ^14^N/^15^N. The balance groups have a mass ranging from 28–31 Da (for the comparison of four samples and from 184–192 Da for eight samples) to compensate the mass difference of reporter groups and identical chemical reactivity and conserve a constant tag mass of 145 Da and 305 Da, respectively. *In fine*, the tags present identical physicochemical properties during the chromatographic step and the MS measure but are distinguished in MS/MS mode. The identification and quantification of the peptides require the fragmentation step in the MS/MS mode. The fragment ions of the reporter groups are detected by their distinctive mass from 114–117 Da (four samples) or 113–121 (eight samples) and their intensities are used for quantification.

The *tandem mass tags* (TMT) labelling is based on the same concept as iTRAQ labelling but differs by the reporter and balance groups. Briefly, the mass of the reporter groups ranges from 126–131 Da, that of the balance group from 99–104 Da while the total mass remains constant (230 Da). The TMT-based reagents allow the simultaneous analysis of two or six samples. The labelling on cysteine residues is also possible [107].

A dimethyl labelling strategy, by reductive methylation using water-soluble formaldehyde, deuterated (CD_2_O) or not (CH_2_O), was developed in 2005 for the binary labelling on the peptide N-terminus and the ε-amino groups of Lys of two sample sets [108,109]. The enzymatic digestion is carried out before the labelling with Lys-C endoproteinase in order to obtain a double labelling per peptide, one at the carboxyl-terminal lysine and one at the amino-terminal group, and a sufficient mass shift (ΔDa = 4 Da) to be detected in MS. The drawback of this technique is that the peptides with a blocked N-terminus (proline, pyroglutamate and so on) are monomethylated and thus poorly detected in MS. This labelling is rapid, inexpensive and specific and leads to almost full reaction. A quadruplex version of this labelling strategy, combining the binary isotopic reagents of formaldehyde (CH_2_O/CD_2_O) and the binary isotopic reducing reagents, sodium cyanoborohydride (sodium cyanoborohydride, NaBCNH_3_ and sodium cyanoborodeuteride, NABCND_3_), was reported one year later [117]. The reagents are combined two by two as follows: CH_2_O/NaBCNH_3_, CH_2_O/NaBCND_3_, CD_2_O/NaBCNH_3_ and CD_2_O/NaBCND_3_ (ΔDa = 4, 8 or 12 Da). However, this dimethyl multiplexed labelling increases the complexity of the analyses. 

The labelling techniques by peptide acylation of free amino groups using stable isotopes, termed *global internal standard technology* (GIST), leads to the uniform labelling of all the peptides. Numerous reagents can be used, such as acetic anhydride, succinic anhydride, *N*-acetoxy-succinimide, 1-nicotinoyloxy-succinimide, propionate, propionic anhydride, 4-trimethylammonium butyrate, isocyanate and isothiocyanate [118,119]. Again, the labelling is performed with reagents containing stable isotopes of hydrogen or carbon. The acylation can however affect the charge of the peptides and, thus, their ionisation. In order to simplify the MS-signal treatment, only the amino-terminal group of the peptides can be tagged (implicates the protection of the ε-amino groups of lysine residues by guanidinylation).

####### 3.3.1.2.2.3. Labelling of Carboxyl Groups

The enzyme-catalysed ^18^O-labelling is known as *enzyme mediated oxygen substitution* (EMOS) [110,111,112]. This labelling, performed late in the sample preparation process, takes place during the enzymatic digestion and acts on the generated α-carboxyl terminus. In the presence of ^16^O-enriched water (H_2_^16^O) or ^18^O-enriched water (H_2_^18^O) or combination of both, the carboxyl terminus will be found as C^16^O^16^OH, C^16^O^18^OH or C^18^O^18^OH depending on the enzyme used (ΔDa = 4 Da or only one ΔDa = 2 Da). The labelling being rarely total, the peptides incorporate the label at various rates, which complicates the analysis. The labelling takes place during the enzymatic digestion with H_2_^18^O or just after the digestion with an additional step of incubation in H_2_^18^O [113]. The challenge of this type of labelling lies in the optimisation of the experimental protocols to avoid residual proteolytic activity creating back ^18^O/^16^O exchanges. The inhibition of the residual, harmful enzymatic activity can be performed by buffer acidification, temperature increase and using trypsin immobilised on beads that can be removed by centrifugation [120]. 

A method of acid-catalysed ^18^O-labelling of proteins/peptides was recently suggested as an alternative to enzyme-catalysed ^18^O-labelling [114]. This *acid mediated oxygen substitution* (AMOS) labelling was applied to a set of peptides after the enzymatic digestion step via chlorhydric acid (HCl) catalysis in the presence of H_2_^18^O to label the carboxyl groups from glutamic acid, aspartic acid, and the C-terminal residues [121]. The optimisation of this labelling allows a 95%–97% incorporation of the tag. Thanks to this method, the majority of peptides display a difference of at least 4 Da limiting the overlapping of isotope patterns of the pair-wise compounds. The possible residual enzymatic activity from the previous step is inhibited by the HCl. The method is not applicable to peptides bearing an acidic pH sensitive PTM. 

Methanol (CH_3_OH) and deuterated methanol (CD_3_OH), in the presence of HCl are used for the esterification of carboxyl groups of aspartate, glutamate and carboxyl terminus [115]. This labelling perfectly fits to the quantification of phosphopeptides, since esterification reduces the undesirable affinity for *immobilised metal affinity chromatography* (IMAC) of acidic peptides, thus improving the yield of fixation of phosphorylated peptides. However, the experimental conditions promote the oxidative deamidation of asparagine and glutamine, which becomes aspartic and glutamic acids, ΔDa = 1 Da) and complicate the data analysis.

####### 3.3.1.2.2.4. Labelling of PTMs

The diversity of PTMs and of their mechanisms of regulation considerably increases the complexity of the proteome. The study and the quantification of proteins with PTMs can be done with isotope labelling using two strategies depending on the target of the labelling: the peptide or the PTM. The first strategy involves the isotopic labelling of all the peptides (amino or carboxyl groups during proteolysis) then the purification of peptides bearing a PTM of interest. For example, the phosphopeptides (phosphoryl groups mainly on tyrosine, serine and threonine residues) are isolated by immobilised metal or metal oxide affinity chromatography (IMAC or MOAC) or using antibodies (for review see [122]). Likewise, the Lys-acetylated peptides are also enriched by immunoprecipitation with antibodies directed against acetyl epitopes. A recent report provides an overview of the recent advances in MS based glycoproteomic methods and technology, in the context of biomarker discovery and clinical application [123]. The glycopeptides can be isolated by lectin-affinity chromatography, by hydrophilic interaction chromatography that can equally carried out by the use of functionalised magnetic beads [124]. However, it is important that the label does not hinder the process of PTM enrichment neither the acquisition of characteristic MS-signals [118]. If that happens, the proteins or peptides with PTMs should first be selected before performing the labelling. The second strategy involves direct targeting of the PTM (phosphoserine or phosphothreonine residues, or glycosylation sites) and their replacement by a reporter group. Briefly the modifications (phosphoryl groups or glycans) are chemically or enzymatically cleaved (β-elimination under mild alkaline conditions, hydrazinolysis, PNGase F, *etc.*) and concomitantly replaced by a reporter group, e.g., 1,2-ethandithiol or its deuterated derivative [125]. The reagent used for the *phosphoprotein isotope-coded affinity tag* (PhIAT) technique includes a biotine residue to easily purify the labelled, initially phosphorylated, peptides from the unlabelled, initially non-phosphorylated ones using an immobilised-avidin inert support [126]. 

#### 3.3.2. Absolute Quantification

For a long time, the reference method for the absolute quantification of proteins was the immunoenzymatic assays, such as the *enzyme-linked immunosorbent assay* (ELISA). However, this is a targeted method, as it addresses the quantification of a unique protein, and has major limitation with regards to the availability and cost of antibodies but also the cross-reactivity of antibodies and the inability to automate. Consequently, new methods, based on mass spectrometry, emerged.

The addition of a protein in known quantities to a sample can lead to an estimation of the absolute abundance of almost all the proteins present in a sample when a *label-free* approach is used. In practice, the final concentration of the supplemented protein is correlated to the MS-signal intensity of peptides issued of its enzymatic digestion and also compared to the MS-signals of all the peptides in the sample. However, this technique leads to significant errors in quantitative ratios and, thus, the interpretation of the results should cautiously be done.

A second method of absolute MS quantification of proteins is based on the use of an internal standard labelled with stable isotopes. It only concerns the quantification of one or a few proteins of interest and therefore it is not adapted to global analyses of a proteome. In fact it is used for studies based on one or a few known proteins for validation of biomarkers in clinical proteomics.

In this context, we can quote the *absolute quantification of proteins* (AQUA) strategy [127], based on the use of a peptide of interest as an internal standard. The selected peptide issued from the protein to be quantified and which is frequently observed in MS is *de novo* synthesised with one amino acid bearing one or more isotopes. Bioinformatics tools help to predict the “ideal” peptides that can serve as internal standards. The physical properties (size, charge, hydrophobicity, and ionisation) of the AQUA peptide remain identical to the native peptide. Their mass difference is observable by MS. The biological sample to analyse is supplemented with a known quantity of the AQUA peptide before enzymatic digestion and LC-MS analysis. A ratio is calculated by the comparison of the mass signal of the native peptide and the AQUA peptide. In the same manner the protein of interest can be directly used as an internal standard and to be added before any treatment of the sample. This technique is known as *protein standard for absolute quantification* (PSAQ) [128]. Both techniques, in theory, enable the study of PTMs, the limit being the relative high cost of the synthesis of isotope-labelled peptides (or proteins) to use as internal standards. 

The quantification of a group of proteins of interest using the AQUA strategy is complex as each standard peptide would need to be chemically synthesised. This can be overstepped with the multiplexed absolute quantification using artificial proteins of concatenated signature peptides (QconCAT). In this method, a unique signature peptide was selected for each protein of interest. The sequences of all the selected peptides are then used to design *de novo* a chimeric gene that expresses the concatenated protein in an expression system that allows its labelling with stable isotopes. The expressed chimeric QconCAT protein is then purified and added to the sample. The enzymatic digestion of the sample containing the QconCAT protein generates peptides including a set of isotope-labelled standard peptides that are used as internal standard [129]. In contrast to AQUA, the PSAQ and QconCAT approaches take into account the process of enzymatic digestion in their quantification principle.

The *selected reaction monitoring* (SRM) and *multiple reaction monitoring* (MRM) overcome certain bias of the standard AQUA method. The MS-analyser (triple-quadrupole) used in these quantification methods controls simultaneously the molecular mass of the intact peptide and of a selected fragment ion (SRM) or of several selected fragment ions (MRM), specific of the peptide. In the first quadrupole, the *m/z* of the peptide of interest is filtered. In the second quadrupole, the selected ion is fragmented using collision energy. Only the fragment ions of interest enter the third quadrupole and thereafter their intensities measured [130]. The concentration of the targeted peptide is then determined by comparison of its mass signals to the mass signals either of the isotope-labelled peptide which was added in known quantities or using a calibration curve. Once the optimisation is completed, the SRM and MRM techniques offer both high sensitivity and reproducibility [131]. SRM is performed in triple quadrupole instruments, although pseudo SRM/MRM experiments are also possible in Q-TOF, Q-orbitrap or also IT instruments [132,133].

### 3.4. Advantages and Limits of the Peptide-Based Quantification of Proteins

Whatever the type of spectrometer used, the *label-free* differential proteomics approach, based on the comparison of the protein heterogeneity of distinct samples is without doubt (i) the less expensive and the less sample-consuming, (ii) the only approach with no limit to the number of experiments to be compared, (iii) well adapted to samples with low complexity, such as the cellular sub-proteomes, (iv) the most accurate in terms of protein identification, however, (v) one of the least quantitative and (vi) the most restrictive concerning the normalisation of the chromatographic separations and their analysis by means of MS (Table 4) [73]. So, compared to stable isotope labelling, the label-free approach is less adapted to the relative quantification of a protein’s abundance between two samples. However, unlike stable isotope labelling, the mass spectra display lower complexity due to the non-simultaneous presence of labelled and unlabelled peptides that allows easier spectral analysis and provides higher dynamic range of quantification.

Concerning the stable isotope labelling, the literature survey reveals several drawbacks. The methods allowing the quantification by MS involve a minimal mass-shift introduced by the labelling. It should be easily detectable by MS and thus distinguish the isotopic pattern of both labelled and unlabelled peptides. It should be noted that the resolution of the isotope patterns decreases inversely proportional to the peptide mass, which means that labelling resulting in low mass-shift will be inadequate for analysis of large peptides when using the most common mass spectrometers. In addition, the mass signals of low intensity (within the background noise) or of high intensity (detector saturation) will not allow a correct quantification. Nevertheless, the development of MS apparatuses and the contribution of the high resolution enable minimising the loss of information. Note that a wrong quantification can always occur, even when high resolution mass spectrometers are used. Indeed, whatever the labelling, the isotopic distribution of the most abundant peptide can completely mask the isotopic distribution of associated peptide.

**Table 4:** Different methods used for the labelling of proteins or peptides in view of off-gel quantification(Peptide based quantification) ^a^.

			Advantages	Drawbacks	Robustness for large scale analysis	Exemples of use
**Pre-analysis labelling** (A single analysis (sample combination after labelling): mass difference between peptide pairs on the same mass spectra)	***In vitro*/*vivo* labelling** (during protein synthesis)	^15^N, ^13^C,	Accuracy	Limited to cells in culture,time-consuming	Yes (regardless of the cost and time needed)	Cells in culture
		SILAC, CIDTs, superSILAC, SILAM				
	**Pre-digestion labelling**	ICPL, iTRAQ, TMT, ICAT, ALICE, dimethyl	Sample complexity	Low sequence recovery		All types of biological sample (regardless of the protein quantity needed)
	**In-digestion labelling**	H_2_^18^O	Simplified signal analysis, low cost	Late labelling		
	**Post-digestion labelling**	ICAT, iTRAQ, ICPL, ALICE, TMT	High sequence recovery			
		dimethyl, GIST				
**Direct analysis** (Two (or more) analyses carried out independently before their comparison)	**Label-free Quantification** (without internal standard)	Comparison of mass spectra	Sample number, low cost	Separations normalisation, signal alignment	Yes	All types of biological sample
		Spectral counting				
	**Absolute Quantification** (with internal standard)	AQUA	Easy to use	Cost of internal standard, analysis of one or few proteins	No(too expensive)	Validation of biomarkers
		QconCAT	Enzymatic digestion take into account			
		PSAQ				
		SRM / MRM	High sensitivity and reproducibility			

^a^ SILAC, stable isotope labelling by amino acids in cell culture; CDIT, culture-derived isotope tags; SILAM, stable isotope labelling of mammal; ICPL, isotope-coded protein label; TMT, tandem mass tags; ICAT, isotope-coded affinity tags; iTRAQ, isobaric tags for relative and absolute quantification; ALICE, acid-labile isotope-coded extractants; GIST, global internal standard technology; Coomassie brilliant blue; XIC, extracted ion chromatogram; AQUA, absolute quantification of proteins; QconCAT, absolute quantification using artificial proteins of concatenated signature peptides; PSAQ, protein standard for absolute quantification; IMAC, immobilised metal affinity chromatography.

Nevertheless, the major limit of the use of stable isotope labelling is associated with the possible co-elution of peptides with other molecules having the same mass. Reducing the complexity of the samples is a prerequisite for the optimisation of the quantitative analyses in this case.

The later the labelling step is performed in the analytical process, the more essential is an expert, as is a rigorous sample preparation and an efficient bioinformatics and statistic treatment of the data. This is particularly true for label-free quantification [73]. In order to avoid inter-sample variations due to experimental bias and to control the reproducibility of the analyses, the number of steps for the sample preparation should be minimised. Other technical bias can occur, especially those resulting from an incomplete incorporation of the isotope or from its low purity.

However, despite the increasing performance of mass spectrometers, the number of identified proteins is limited compared to the high number of proteins in a given sample along with the employed methodology. The number of quantified proteins will inadvertently be different to the identified proteins, since all the proteins are not always present in the sample under consideration. The identification and quantification ratios are thus directly related to the sample complexity. 

The last step of a proteomics analysis, not detailed in this review, but essential, relates to the verification of the results. This step is as equally important as the generated data. The reliability of the obtained results is indeed linked to the biological conclusions issued from the analysis. The results can be subjected to statistical analysis, error rate assessment [134], to manual checking (data processing) and be also validated by further biochemical analyses. A recent review [135] highlighted important issues that directly impact the effectiveness of proteomic quantification and educates software developers and end-users on available computational solutions to correct for the occurrence of these factors; potential sources of errors specific for stable isotope-based methods or label-free approaches are explicitly outlined.

## 4. MS Technology for Proteomics

The MS technology emerged at the beginning of the 20th century, essentially in response to the need for detecting and quantifying atoms. Due to their high molecular mass, the technology was adapted only from around 20 years ago to the analysis of biological macromolecules. The mass spectrometers experienced prodigious improvements in recent years thus contributing to the emergence of the MS-based proteomics even if the MS resolution and detector performance are presently limiting [136]. The choice of the type of mass spectrometers will depend on the retained proteomics strategy, the considered approach and the desired degree of information. Because mass analysis uses electromagnetic fields in a vacuum, molecules must first be electrically charged and transferred into the gas phase. Once in the gas phase, the *m/z* ratio of molecules is measured from their trajectories in a static or dynamic electric field. For example, a quadrupole mass filter can be set to only transmit ions of a given *m/z* and a mass spectrum is then obtained by scanning through a range of *m/z* values. Two types of sources are used for the “soft” ionisation of proteins and peptides: (i) the electrospray ionisation source (ESI) [137,138] a continuous source for the ionisation of compounds in solution directly after their separation in liquid phase (chromatography or electrophoresis) or in gas phase (gas liquid chromatography); and (ii) the *Matrix-assisted laser desorption/ionization* (MALDI) source [139,140,141], a non-continuous source (ionisation by energy pulse) in solid phase (the compounds are previously co-crystallised in a matrix), which is based on the desorption of molecules triggered by UV laser beam and their consecutive ionisation.

The mass analysers used for proteomics approaches can be more or less complex—quadripoles (Q), time of flight (TOF), *ion traps* (IT), Fourier transform-IT (*Orbitrap*^TM^) and Fourier transform ion cyclotron resonance (FT-ICR)—all individually having their own advantages and/or drawbacks (for review, [10,142]. They can be used in MS mode for the accurate measure of the *m/z* ratio or combined each other (MS/MS mode or tandem MS) for sequencing of the peptides by physical fragmentation. The sensitivity, the resolution, the mass range, the rate of scanning and the precision are the parameters to be specified for the mass analyser.

In the analysis of peptides derived from the same protein, typically after 2-DE, the identification of the protein will be done mainly by the establishment of PMF of the protein after the action of a specific protease, using the MS mode, which measures the *m/z* ratios of generated peptides. Proteases with restrained specificity always generate the same peptides for a given protein [143,144,145,146,147]. However, it may be appropriate to combine PMF and PFF results in order to avoid false positive and to increase identification scores. In the analysis of peptides derived from a mixture of proteins, typically after HPLC, the identification will only be done by establishment of the PFF via the MS/MS mode, which measures the *m/z* ratios of the different fragments of the peptide [148,149,150]. The information on the masses obtained by PMF or PFF is then compared to their putative homolog predicted *in silico* from the sequences of all the proteins referenced in the protein data banks [151] or from any DNA/RNA databank. Those comparisons are performed using dedicated software such as Profound, MS-FIT, Mascot, Sequest, X!Tandem or OMSSA [152]. The close match of the theoretical and the experimental PMF (or PFF) allows tracing back the protein identity. Developed for the identification of proteins whose sequences are already present in the data banks, the aforementioned methods do not allow the identification of proteins derived from non-sequenced organisms, unless by their homology to existing sequences, or, proteins containing complex PTMs. For those proteins (or peptides) the MS/MS mode allows *de novo* sequencing and, thus, helps deducing their amino acid sequence from the mass difference between consecutive ion fragments. In that case, it becomes possible to identify peptides with PTMs and those originating from non-sequenced organisms (due to species homology).

## 5. Concluding Remarks

Given the continuous developments of quantitative strategies (especially the isotopic labelling methods) and MS apparatus and the different challenges in proteomics, it is difficult to describe exhaustively, even succinctly, all the techniques and the possible strategies for the quantification of proteins. Thus, this review was focused on the well-established techniques, while keeping in mind that techniques are modular and often overlap each other. Considering the unique characteristics and limitations of each approach and the diversity of the physicochemical properties of proteins, all approaches discussed here are considered complementary with each other. 

The overview highlighted the advantage of global proteomics strategies, which is that no hypothesis is required, other than a measurable difference in one or more protein species between the samples. Since global proteomics methods attempt to separate, quantify and identify all the proteins from a given sample, the *bottom-up* strategy is clearly of choice for a global differential study of proteins. In the effort of deciphering molecular mechanisms for the establishment of the blood-brain barrier, we have experimented with several approaches and, using the *in vitro* model developed in our laboratory, we demonstrated that the in-gel [50,153] and the off-gel [45,49,50] approaches were complementary. However, the in-gel approach seems to be the approach of choice to initiate a comparative and quantitative global proteomic study of an “unknown” sample, whereas the off-gel approach allows going deeper in the analysis once the identity of a sample has been established. Thus, this is more than ever noteworthy that the “choice of an *in-gel* or an *off-gel* analysis as well as the choice of the quantification strategy to use will only depend on the biological question we have to tackle” [5].

To conclude, the label free approach seems to be the approach of choice in the future because of (i) a real progress in MS-instruments (higher mass accuracy and faster scanning), computational methods and software for data treatment and (ii) its low cost compared to labelling approaches. For comprehensive characterization of proteomes, an analytical platform capable of quantifying protein abundance, identifying post-translation modifications and revealing members of protein complexes on a system-wide level is necessary. MS, coupled with technologies for sample fractionation and automated data analysis, provides such a versatile and powerful platform [154]. Understanding protein interactions within the complexity of a living cell is challenging, but techniques that combine affinity purification and MS have enabled important progress in recent years. The quantification of the interaction dynamics is the next frontier. Several quantitative mass spectrometric approaches have been developed to address these issues that vary in their strengths and weaknesses [155]. While isotopic labelling approaches continue to contribute to the identification of regulated interactions, label free techniques are becoming increasingly used in the field, as was recently done for the study of *N*-glycan occupancy in *N*-glycoproteins [156].
